# Did COVID-19 impact stroke services? A multicenter study

**DOI:** 10.1007/s10072-022-06018-7

**Published:** 2022-03-25

**Authors:** Hossam Shokri, Nevine El Nahas, Ahmed El Basiony, Thanh N. Nguyen, Mohamad Abdalkader, Piers Klein, Pablo M. Lavados, Verónica V. Olavarría, Pablo Amaya, Natalia Llanos-Leyton, Waldemar Brola, Lipowski Michał, Donoband Edson Dejesus Melgarejo Fariña, Analia Cardozo, Cesar David Caballero, Fatima Pedrozo, Aminur Rahman, Elyar Sadeghi Hokmabadi, Javad Jalili, Mehdi Farhoudi, Hany Aref, Tamer Roushdy

**Affiliations:** 1 Faculty of Medicine, Ain Shams University, Cairo, Egypt; 2 Neurology Department, Faculty of Medicine, Ain Shams University, Cairo, Egypt; 3 Boston Medical Center, Boston University School of Medicine, Boston, USA; 4 Unidad de Neurología Vascular, Servicio de Neurología, Departamento de Neurología Y Psiquiatría Facultad de Medicina Clínica Alemana, Universidad del Desarrollo, Santiago, Chile; 5 Stroke Program, Neurology Department, Fundación Valle del Lili, Cali, Colombia; 6 Clinical Research Center, Fundación Valle del Lili, Cali, Colombia; 7 Department of Neurology, Specialist Hospital Konskie, Collegium Medicum, Jan Kochanowski University, Kielce, Poland; 8 Stroke Unit of the Instituto de Previsión Social Central Hospital, Asunción, Paraguay; 9 Sir Salimullah Medical College, Dhaka, Bangladesh; 10 Neurosciences Research Center (NSRC), Tabriz University of Medical Sciences, Tabriz, Iran; 11 Tabriz University of Medical Sciences, Tabriz, Iran

**Keywords:** Acute stroke services, Hemorrhagic stroke, Stroke data base, Pandemic-related issues

## Abstract

**Background:**

It has been reported that acute stroke services were compromised during COVID-19 due to various pandemic-related issues. We aimed to investigate these changes by recruiting centers from different countries.

**Methods:**

Eight countries participated in this cross-sectional, observational, retrospective study by providing data from their stroke data base. We compared 1 year before to 1 year during COVID-19 as regards onset to door (OTD), door to needle (DTN), door to groin (DTG), duration of hospital stay, National Institute of Health Stroke Scale (NIHSS) at baseline, 24 h, and at discharge as well as modified Rankin score (mRS) on discharge and at 3 months follow-up.

**Results:**

During the pandemic year, there was a reduction in the number of patients, median age was significantly lower, admission NIHSS was higher, hemorrhagic stroke increased, and OTD and DTG showed no difference, while DTN time was longer, rtPA administration was decreased, thrombectomy was more frequent, and hospital stay was shorter. mRS was less favorable on discharge and at 3 months.

**Conclusion:**

COVID-19 showed variable effects on stroke services. Some were negatively impacted as the number of patients presenting to hospitals, DTN time, and stroke outcome, while others were marginally affected as the type of management.

## Introduction

COVID-19 was declared as a pandemic in March 2020, and since then, emergency hospitalization for stroke and cerebrovascular disease has been reportedly reduced [1–5].

This reduction might have been attributed to stay-at-home and social distancing recommendations by health authorities in different parts of the world and to the fear of public from contracting COVID-19 at hospitals [6–8].

During the pandemic, the severity of stroke among patients presenting for care seems to have increased. This could be ascribed to patients with milder severity of disease refraining from going to hospitals, either because emergency departments were overwhelmed with COVID-19 cases or due to the fear of patients contracting infection at hospitals [9].

In addition, there was a controversy among different reports regarding the change of pattern of acute stroke management [10] that resulted in either an increased, decreased, or no change of onset to door or door to needle times [11]. Consequently, the outcome of stroke has changed to variable degrees in different health facilities [4].

Since the onset of this pandemic, the world has been confronted by several waves and is expected to face more waves that can further compromise stroke health care. In this study, we explored if there was any change in the quality of acute stroke services during the COVID-19 period compared to a similar period pre-COVID-19. We also studied the clinical picture and patient outcome across the two time periods. Unveiling any setbacks in stroke management can assist health authorities to take appropriate measures during any coming pandemic waves.

## Methods

This is an observational, retrospective, multicenter study assessing the quality of care provided to patients admitted to various stroke centers. More than 400 stroke centers from 70 countries that actually collaborated in a previous study [5] were contacted via e mail 3 times (by author H. S.) with 45 days periods to prepare the data. Ten centers responded, and 2 centers were excluded due to incomplete data. The participating centers are from 8 countries: Iran, the USA, Egypt, Poland, Chile, Colombia, Bangladesh, and Paraguay. Assessment spanned 2 consecutive periods of equal duration: 1 year (March 2019–February 2020) pre- and 1 year (March 2020–February 2021) post-declaration of the COVID-19 as a pandemic by the World Health Organization (WHO).

Participating countries ought to have the following capabilities as prerequisites for participation: a stroke center providing thrombolysis and thrombectomy services, in addition to a database fulfilling the following information: onset to door (OTD), door to needle (DTN), door to groin (DTG), duration of hospital stay, National Institutes of Health Stroke Scale (NIHSS) recorded at baseline, 24 h, and discharge as well as modified Rankin score (mRS) on discharge and at 3 months follow-up.

The year preceding declaration of COVID-19 as a pandemic was compared with the post-declaration year for variables reflecting quality of care and service provided: DTN, DTG, duration of hospital stay, and frequency of administration of rtPA and thrombectomy.

Indirect variables that assess the quality of care included change across the two time periods, in scores of stroke severity (NIHSS at onset), in addition to outcome scales including NIHSS at 24 h and at discharge, as well as mRS scores on discharge and 3 months follow-up.

Factors reflecting the effect of the pandemic on human behavior of seeking medical advice for an urgent medical emergency (Stroke) were also compared between the 2 years through OTD and NIHSS at presentation.

## Statistics

Statistical analysis was done using SPSS version 19th version Statistics (SPSS Inc., Chicago). To test for normality of continuous data distribution, the Shapiro-Wilks test was used. Mean and standard deviation were used for normally distributed data, while median and interquartile range (IQR) were used for skewed data. Categorical data were presented as frequencies. Mann–Whitney Test used to compare not normally distributed continuous variable with nominal independent variables. The chi-square test was used for comparison of nominal data.

## Results

The total number of patients included in this study is 5313, recruited from eight countries: Iran, the USA, Egypt, Poland, Chile, Colombia, Bangladesh and Paraguay **(**Table 1**)**. The clinical characteristics of the whole sample are shown in Table 2.

**Table 1:** The number of patients recruited in each center and their demographics

	Iran	USA	Egypt	Poland	Chile	Colombia	Bangladesh	Paraguay
No. of patient recruited in each center	1519	1224	1174	579	272	260	168	117
Age, median (minimum- maximum)	69 (13–102)	67 (7–101)	63 (20–98)	73 (32–92)	74 (21–101)	72 (22–103)	56 (31––85)	69 (33–89)
Male gender, %	53.9	51.1	60.9	58.7	57	50.4	54.8	68.4
Hypertension, %	70.8	68	61.1	88.9	59.9	66.5	97	90.6
Diabetes mellitus, %	24.1	24.6	46.3	52.8	16.2	24.2	60.1	35.9
Atrial fibrillation, %	5.7	16.7	7.8	58	21.3	20	28.6	16.2
Previous stroke, %	17.2	17.6	13	9.8	19.9	15.8	21.4	13.7

**Table 2:** Clinical characteristics of the total sample and comparison between clinical characteristics of patients pre- and during COVID-19 period:

Variables	Total sample *N* = 5313	Pre COVID-19 *N* = 2795	During COVID-19 *N* = 2518	*p*-value
Age (years)*	66.7 (14)	68 (58–77)	67 (57–76)	< 0.01
Male gender, %	55.6%	55.2%	56.1%	0.54
Management, %				0.054
Standard	78.8%	79.4% (2219)	78% (1965)	
rtPA	15.9%	16.1% (450)	15.8% (397)	
Thrombectomy	2.8%	2.3% (65)	3.3% (84)	
rtPA and thrombectomy	2.5%	2.2% (61)	2.9% (72)	
Onset to door (minutes)*	360 (162–1022)	370 (154–1199)	360 (165–901)	0.161
Door to needle (minutes)*	41 (30–57)	38 (30–49)	45 (30–64)	< 0.01
Door to groin (minutes)*	121 (80–177)	122 (72–180)	121 (91–175)	0.616
Hospitalization (days)*	7 (4–11)	7 (4–12)	7 (3–10)	< 0.01
NIHSS baseline*	9 (4–15)	8 (4–14)	10 (3–16)	< 0.01
NIHSS 24 h*	7 (3–12)	6 (3–10)	8 (4–12)	< 0.01
NIHSS discharge*	6 (2–11)	5 (2–11)	6 (2–11)	0.280
mRS discharge	33%	34.9%	31.1%	0.005
mRS 3 months	40.2%	42.5%	37.4%	< 0.01

mRS, favorable outcome < 2, *median (interquartile range); *p* value, between clinical characteristics of patients over the two time periods

The number of acute stroke patients was 2795 in the pre-COVID and 2518 during COVID, with a drop of 277 cases, representing 9.9% reduction of admitted stroke cases during the pandemic.

The median age was higher in the pre-COVID time than during COVID time (68 and 67 years respectively) (*p* =  < 0.01), but gender did not differ significantly across both periods where males represented 55.2% and 56.1%, respectively.

The type of management displayed marginal, non-significant differences between the two time periods. The frequency of rtPA administration was marginally higher during pre-COVID by (0.3%), while thrombectomy and combined rtPA/thrombectomy showed slight increase during COVID than pre-COVID (3.3% and 2.9%, respectively).

Onset to door and door to groin times showed non-significant difference across the two time periods, while door to needle time was significantly longer during COVID and duration of hospital stay was significantly shorter (*p* =  < 0.01 each).

As for stroke severity by NIHSS, it was significantly higher during than before pandemic at baseline (10 and 8, respectively) and 24 h after admission (8 and 6, respectively) (*p* =  < 0.01), but it showed non-significant difference at discharge (6 and 5, respectively). On the other hand, the frequency of patients with a favorable outcome (defined as mRS < 2) was significantly higher for pre- compared to during COVID patient groups, whether on discharge (34.9% and 31.1% respectively) or at 3 months follow-up (42.5% and 37.4% respectively; *p* = 0.005 and 0.002 respectively) **(**Table 2**)**.

Comparing different countries across time periods, Egypt had an increase of age during COVID, while Iran had a decreased age. Possibly Iran contributed to the significant global decrease of age found in Table 2 as it has the bigger number of patients. The same applies to global decrease of rtPA administration in Table 2 that is found in most countries being significant only in Iran, whereas Egypt and Paraguay had an increase of rtPA during COVID. The door to needle time increased globally and was found also to increase significantly in Egypt and Iran only. Hospital stay decreased significantly in Iran and Chili. NIHSS on admission and discharge increased globally and is significant in Egypt and Iran. The number of patients with good outcome by mRS on discharge and at 3 months generally decreased reaching significant levels only in Egypt and Iran, while Paraguay was the only country having an increased number of patients with good outcome at discharge. These finding reveals a significant difference between countries, which indicate a different impact of the pandemic across the countries **(**Table 3**)**.

**Table 3:** Clinical characteristics and comparison between clinical characteristics of patients pre- and during COVID-19 in each center separately

		Age (years)*	Male gender, %	Management, %				Onset to door (minutes)*	Door to needle (minutes)*	Door to groin (minutes)*	Hospitalization (days)*	NIHSS baseline*	NIHSS 24 h*	NIHSS discharge*	mRS discharge, %	mRS 3 months, %
				Conservative	rtPA	Thrombectomy	rtPA and thrombectomy									
Iran	Pre COVID-19	71 (60–81)	54.1	78.9	21.1	0	0	252 (117–804)	42 (32–54)	-	7 (4–13)	12 (6–20)	10 (4–17)	9 (3–21)	27.6	30.9
	During COVID-19	67 (56–76)	53.5	84.9	14.5	0	0.6	293 (134–788)	47 (38–60)	105 (102–131)	7 (4–11)	14 (6–25)	10 (5–16)	11 (4–40)	25.1	24.8
	p-value	0.00	0.8	< 0.01				0.1	< 0.01	N/A	0.01	0.03	0.8	< 0.01	0.3	0.01
USA	Pre COVID-19	67 (56–78)	47.3	83.9	5.1	7	4.1	608 (210–1702)	48.5 (35.5–71)	74.5 (58–112.5)	5 (3–10)	4 (1–13)	-	-	38	-
	During COVID-19	66 (55–77)	55.9	84.3	2.4	9	4.3	871 (294–2232)	52 (38–78)	94 (52–130)	5 (2–10)	4 (1–15)	-	-	32.7	-
	p-value	0.5	< 0.01	0.07				< 0.01	0.5	0.9	0.3	0.8	N/A	N/A	0.05	N/A
Egypt	Pre COVID-19	63 (55–70)	60.6	82.2	15.8	0.6	1.4	600 (300–1440)	30 (30–30)	-	-	6 (3–10)	5 (3–8)	4 (2–6)	41.3	56.9
	During COVID-19	65 (57–71)	61.3	72.5	25.3	0.8	1.4	360 (150–960)	30 (30–45)	-	-	7 (4–11)	6 (3–10)	4 (2–8)	34.4	49.8
	p-value	< 0.01	0.8	< 0.01				< 0.01	0.01	-	-	< 0.01	< 0.01	< 0.01	0.03	0.02
Poland	Pre COVID-19	73 (68–79)	60.7	72.5	22.2	0.6	4.8	265 (130–347.5)	40 (30–45)	170 (160–185)	10 (9–12)	12 (11–13)	8 (6–11)	6 (3–7)	32	35.1
	During COVID-19	73 (68–78)	55.6	74.4	20.6		4.9	255 (130–340)	40 (30–45)	180 (167–191)	10 (9–11)	12 (11–14)	8 (6–12)	6 (3–7)	28.9	29.7
	p-value	1	0.3	0.7				0.9	0.9	0.2	0.6	0.4	0.4	0.8	0.4	0.2
Chile	Pre COVID-19	75 (61–83)	56	59	29.9	6.7	4.5	186 (56.5–908.5)	36 (25–56)	84 (70–120)	4 (3–8)	3 (1–7)	2 (1–5)	1 (0–3)	58.1	69.1
	During COVID-19	73 (62–82)	58	59.4	24.6	5.8	10.1	195.5 (85.7–803)	38 (26–60.5)	108 (77–139)	3 (2–6)	3 (1–10)	3 (0–7)	0 (0–3)	58.1	64.3
	p-value	0.8	0.7	0.3				0.4	0.9	0.2	< 0.01	0.8	0.5	0.2	1	0.4
Colombia	Pre COVID-19	-	-	-	-	-	-	-	-	-	-	-	-	-	-	-
	During COVID-19	72 (62–81)	50.4	75.4	10.4	9.2	5	274 (152–681)	65 (47.5–84)	127.5 (103–231)	4 (2–8.8)	6 (2–15.8)	5 (1–16)	2 (0.8–7)	43.8	51.8
	p-value	N/A	N/A	N/A				N/A	N/A	N/A	N/A	N/A	N/A	N/A	N/A	N/A
Bangladesh	Pre COVID-19	-	-	-	-	-	-	-	-	-	-	-	-	-	-	-
	During COVID-19	56 (50–63)	54.8	73.8	26.2	0	0	360 (180–480)	130 (120–180)	-	7 (5–8)	16 (15–18)	12 (12–15)	10 (8–10)	0	0
	p-value	N/A	N/A	N/A				N/A	N/A	N/A	N/A	N/A	N/A	N/A	N/A	N/A
Paraguay	Pre COVID-19	67 (59–76)	67.3	90.9	5.5	3.6	0	720 (300–2880)	-	-	11 (8–14)	7 (3–10)	6 (2–10)	5 (2–10)	18.2	-
	During COVID-19	69 (61–76)	69.4	72.6	27.4	0	0	360 (204–1440)	45 (30–60)	-	13 (9–17)	7.5 (4–15)	6.5 (3–13.5)	4.5 (2–10)	37.1	-
	p-value	0.7	0.8	< 0.01				0.02	N/A	N/A	0.05	0.3	0.5	0.5	0.02	N/A

N/A, not appropriate; -, not available; mRS, favorable outcome < 2, *median (interquartile range); *p* value, between clinical characteristics of patients over the two time periods

Stroke subtypes did not differ in the two time periods except for intracerebral hemorrhage that showed a significant increase during COVID reaching 11.2% compared to 8.5% pre-COVID, (*p* = 0.01) **(**Table 4**)**.

**Table 4:** Types of stroke pre- and during COVID-19 period

Type of stroke	Pre-COVID-19	During COVID-19	*p* value
Ischemic	87.7%	84.5%	0.01
TIA	0.6%	0.8%	
Intracerebral hemorrhage	8.5%	11.2%	
Subarachionid Hemorrhage	3.1%	3.5%	

*TIA* transient ischemic attack

## Discussion

In this study we reviewed stroke patients from different parts of the world to evaluate any change in the pattern of stroke presentation, management and outcome over a 2-year period, namely, before and during COVID-19.

We found a 9.9% reduction in the number of cases admitted during the COVID-19 period. This is less than that reported by Tong et al. 2021 and Diegoli et al. 2020, where they found a 20.2% and 36.4% reduction, respectively [12, 13].

Patients admitted during the pandemic were significantly younger. It is possible that older patients, especially those living alone, were inaccessible to rescue either through family members or medical services due to lock-down regulations implemented at the time of pandemic [13–15].

It was also observed that onset to door time did not change, possibly the curfew and lock down made transportation to hospital easy for patients and caregivers [16].

Of note, door to needle time was significantly longer during the COVID pandemic which was also observed in another multicenter study [17]. The same observation was previously attributed to in-hospital implementation of new protocols for patient triage due to the pandemic [18].

In contrast, the in-hospital services seemed to be unaffected by the pandemic in several other centers as the door-to-needle time for rtPA remained unchanged [7, 13, 19–24].

Although the absolute frequency of rtPA administration decreased during the pandemic, when calculated as a percentage of the total number admitted, the percentage of decline was 0.3%. Similarly, other reports stated that the ratio of reperfusion therapies to total stroke admissions was maintained during the pandemic [25]. It was thus concluded that the reduction in reperfusion therapies resulted from the diminished number of stroke patients reaching hospitals [23, 26].

We found that the door to groin time did not change and that the frequency of thrombectomy tended to increase by 1% during pandemic. Our findings coincided with others [24, 25] who showed that door to groin time did not change and that thrombectomies were done in due time possibly because routine activities were cancelled; thus specialized personnel were readily available for emergency stroke interventions [18].

Nevertheless, some studies reported delays in treatment workflow for acute stroke within hospitals. Some centers suffered from a decrease in the frequency of reperfusion therapies as well as an increased time to therapy [27–30].

As for the subtype of stroke, there was a significant increase in hemorrhagic stroke during the pandemic, while the ratio of TIAs to ischemic strokes did not change. We postulate that the increased use of anticoagulation therapy during COVID-19 or the lack of control of vascular risk factors due to decline of routine health services could have contributed to the increase in hemorrhagic strokes [31].

On the other hand, the reluctance of patients to reach for medical services was reflected on significantly higher NIHSS scores and lower number of patients with mild strokes. Similar findings were reported with an additional increase of in-hospital death rates from 4.3 to 5% [13].

Another etiology for the increased stroke severity and worse outcome was attributed, by a systematic review, to increased stress and depression during the pandemic. Psychosocial stressors have been associated with more severe strokes and poor outcome [32, 33].

The increased severity of stroke on admission was reflected on patient outcome. The number of patients with favorable outcome was significantly less during COVID whether at discharge or at 3 months follow-up. Contrary to our findings, other studies found no significant difference in the frequency of mRS (0–2) at discharge between the two periods [34, 35].

When we compared different countries, we found variable effects of COVID on stroke. Although the global effect might seem to be in the direction of decline in some variables like administration of rtPA, yet some countries reported an increased frequency of administration. This discrepancy between the global and differential results can be attributed to the bigger statistical weight of some countries as they participated with a bigger number of patients. Also, some countries as Bangladesh and Colombia only started their stroke registries during COVID, so they could not provide pre-COVID data. Thus, they were merged in the global data as cases during COVID but were not subject to within group analysis.

## Conclusion

In this multicenter study of eight countries, COVID-19 has negatively impacted some aspects of stroke care. Patients presenting to hospitals had severe strokes together with a prolonged DTN time both of which might explain the less favorable short- and long-term outcomes. While other aspects showed only a marginal change.

### Study limitations

Although most of the variables studied differed from pre- to during COVID, yet some of these differences were marginal, and even those that were statistically significant need verification by further studies to investigate their clinical relevance.
